# Differential Temporal Shifts in Skin Bacteria on Wild and Captive Toads

**DOI:** 10.1007/s00248-025-02537-w

**Published:** 2025-04-29

**Authors:** Chava L. Weitzman, Kimberley Day, Gregory P. Brown, Karen Gibb, Keith Christian

**Affiliations:** 1 Research Institute for the Environment and Livelihoods, Charles Darwin University, Casuarina, NT Australia; 2 School of Natural Sciences, Macquarie University, Sydney, NSW Australia

**Keywords:** Cane toad, Captivity, *Rhinella marina*, Skin microbiome, Stability, Temporal dynamics

## Abstract

Skin bacteria on amphibian hosts play an important role in host health, but those communities are also constantly shifting based on environmental and host-related feedback. On some hosts, stability of skin communities depends on relatively abundant taxa, with less abundant taxa more readily entering and exiting the system. Cane toads (*Rhinella marina*) have invaded widespread, diverse tropical ecosystems, with varying ecology, physiology, and behaviour in different environments. In this study, we described temporal patterns of skin bacterial communities on cane toads at a site in northern Australia through the wet and dry seasons over two years. Toads in the wild population were paired with a captive-held population, housed in a semi-natural environment, to detect effects of time and season on wild toads, explore bacterial transience and volatility in skin taxa, and determine the extent to which skin communities on captive toads represent those on the wild population. We found community differences by captivity status, sampling timepoint, and season, with increased richness in the wet season on wild toads. Bacterial communities also became more similar among individuals (lower dispersion) in the wet season. Captive toads harboured more stable communities over time, likely owing to the reduced bacterial reservoirs experienced while in captivity. We propose that cane toads, with varied movement patterns among their diverse invaded habitats, provide an interesting direction for future work understanding the influences of habitat and movement on skin microbes, and the flexibility of microbial symbiotic interactions in invasive hosts.

**Supplementary Information:**

The online version contains supplementary material available at 10.1007/s00248-025-02537-w.

## Introduction

Symbiotic bacterial communities in and on organisms play important roles in host health and homeostasis [1, 2]. It may then seem contradictory that bacterial communities are constantly in flux, changing in membership and structure in response to environmental and host cues [3–6]. For example, when exposed to warmer temperatures, gut bacterial communities in lizards experience reductions and shifts in diversity and functional profiles, with possible physiological consequences [7]. In amphibians, skin bacteria experience biomass reductions and community shifts in response to regular, cyclical skin sloughing every few days [6, 8]. Though the roles of bacterial microbiomes are becoming better understood in wildlife systems, there are still many gaps in our understanding of the extent of variation wild individuals experience in their microbiome over time and how that variability interacts with host health.

Amphibian skin bacteria have been widely studied, largely in response to the pandemic fungal disease, chytridiomycosis, affecting countless amphibians worldwide [9]. Field surveys have uncovered many spatial, ontogenetic, environmental, and seasonal patterns of amphibian skin communities [3, 4, 10–12]. Unfortunately, small, motile, wild animals are difficult to follow over time due to recapture difficulty and seasonal reductions in activity making many ectotherms difficult to locate. These issues are compounded with logistical and funding limitations, restricting the scope of many studies and at times replacing temporal replication with spatial replication to detect broader environmental patterns.

In a long-term study of endangered Sierra Nevada yellow-legged frogs (*Rana sierrae*), 19 individual frogs were resampled up to six times over 2 years, revealing important patterns of stability in skin bacteria on wild amphibians [13]. Results of that study found increased stability in communities with lower diversity and reduced transience (i.e. more consistent presence of taxa) in more relatively abundant taxa [13]. Others have described similar findings in captive frogs [14]. Following captive individuals over time can reveal insights about community processes, but changes in captivity and associated changes in environmental bacterial reservoirs can quickly change skin bacteria [5, 15]. Consequently, understanding the extent that communities on captive-held animals represent those on wild populations is an important consideration in mesocosm studies.

In this study, we investigated temporal changes and stability in skin bacterial communities of cane toads (*Rhinella marina*), comparing wild populations with captive-housed toads over two years. Through repeated sampling of skin microbiomes (specifically, bacterial taxa identified from 16S amplicon sequencing) from captive-housed toads and those in the surrounding wild population, we addressed the following hypotheses: 1) Skin bacterial community composition varies over time and by season, reflecting the influence of climatic and weather variables [16, 17]. 2) Temporal patterns of skin microbiomes in captive-housed toads mirror those in wild toads, despite differences in environmental complexity. For example, if richness or dispersion increases in a season, that change would be seen in both wild and captive toads. 3) Captive-housed toads exhibit distinct and more stable skin bacterial communities compared to wild toads, due to reduced exposure to diverse environmental reservoirs [14, 15]. Moreover, we predicted that host body condition would correlate with skin bacterial diversity in wild toads, reflecting their movement and interaction with diverse environments [18]. Additionally, we predicted that bacterial taxa with greater relative abundance would exhibit reduced transience and volatility, contributing to overall community stability [13].

## Methods

### Experimental Design

This study was conducted at Macquarie University’s Tropical Ecology Research Facility in Middle Point, Northern Territory (NT), Australia. Toads at this site experience a wet–dry seasonal, tropical climate, where rainfall and humidity vary widely over the course of a year, with remarkably less variation in maximum temperatures. Unlike many other amphibians in the region, cane toads are relatively active year-round, enabling wet- and dry-season sampling of wild individuals.

In May 2021, 25 wild cane toads were swabbed (see *Sampling* below) and placed into two semi-natural, outdoor enclosures. Captive toads were held in 700 L enclosures with Middle Point soil, artificial lighting above to attract insect prey, and automatic sprinklers to provide water. Tanks were kept on an angle to provide wet and dry options. Before the second sampling date, 11 toads died or disappeared, presumably due to predation. Consequently, the original experimental toads were combined into one enclosure, and 11 additional wild toads brought into captivity to keep the sample size at 25 toads. At this point, wire mesh covers were placed on the tanks to reduce predation. After another mortality event by early 2022, nine more toads were added to the experiment, as well as a third enclosure to reduce toad densities and minimise cannibalism. Similarly, two more toads were added to the enclosures in June 2022, after which no further missing toads (*n* = 3) were replaced. After the third enclosure was added, the three enclosures contained 5–9 toads at a time. Captive toads were toe-clipped for individual identification.

Captive toads were periodically sampled from 2021–2023 (Table 1). On the same dates, we also sampled wild toads hand-collected at Middle Point, allowing a comparison between captive and wild individuals. Wild toads were not released until all had been sampled so that no toad was sampled twice within a time point. We focused the current study on the 15 individuals held in captivity from mid-2021 through the end of the experiment, ensuring at least four repeat samples per captive toad (Table 1). Microbiome samples include those from when the toads were brought into captivity (“intake”) and four time points representing wet (February) and dry seasons (May/June) of 2022–2023, alongside samples from 15 wild toads during the same sampling rounds (Table 1). Captive and wild toads measured 111.6 ± 12.65 and 117.0 ± 14.24 mm snout-urostyle length on average (± SD), respectively (sampled in 2022–2023). The 15 captive toads included in the present study comprised six females and nine males based on secondary sex characteristics and vocalisations (2:3 female:male sex ratio). Wild toad sex ratios were 3:2, 4:1, 2:1, and 3:2 for sampling rounds 3–6, respectively (*N* = 15 per round). Sex was not significant in preliminary analyses and was excluded from analyses below. Wild toads were not permanently marked due to very low recapture rates in the area [19].

**Table 1:** Sample sizes included in the present study. We focused on tracking microbiome communities of 15 captive toads in the dry (May/June) and wet (February) seasons, comparing those experimental captive toads with nearby wild toads. Eight of the captive toads followed were initially captured in May 2021, and the remaining seven were added to the experiment in July 2021. The first sampling of a toad brought into captivity was treated as a wild toad sample, and sometimes referred to as an “intake” sample. ^#^One sample from the captive toads in Feb 2023 failed in sequencing

*Sampling round*	*Captive*	*Wild*
(1) May 2021		15
(2) Jul 2021		7
(3) Feb 2022	14	15
(4) Jun 2022	15	15
(5) Feb 2023	15^#^	15
(6) May 2023	15	15

### Sampling

Each toad was rinsed with 100 mL ultra-pure water and swabbed with a sterile synthetic swab (Copan FLOQSwabs, Copan Diagnostics Inc., Murrieta CA, USA) to collect skin microbes. Swabbing included 30 strokes over all body regions, excluding the face and cloaca [20]. Swabs were preserved in 300 µL Zymo DNA/RNA Shield (Zymo Research, Irvine, CA, USA) and stored at 4˚C until DNA extraction. During sampling, we collected an environmental control, exposing a swab to the ambient air and preserving it for DNA extraction alongside toad samples.

### DNA Extraction and Microbiome Sequencing

DNA was extracted with the Norgen Microbiome DNA Isolation Kit (Norgen Biotek Corp., Thorold, ON, Canada) and quantified with a NanoDrop Spectrophotometer (ThermoFisher Scientific). The 141 toad samples and 4 environmental control samples were diluted to 5–10 ng/μL and sent to Ramaciotti Centre for Genomics at the University of New South Wales for library preparation and sequencing. The V4 region of bacterial 16S rRNA gene was amplified with the Earth Microbiome Project 515 F/806R primers [21–23] and sequenced on a MiSeq v2 with 2 × 250 bp paired-end sequencing.

### Data Processing

In QIIME2 v2024.2 [24], dada2 [25] was used to truncate reads (forward 240 bases, reverse 230 bases), allow a maximum expected error of 5, and join paired ends. Taxonomy of ASVs was assigned with sklearn and a Silva v138 515 F/806R classifier [26–28]. Any reads not assigned as Bacteria, or assigned to mitochondria or chloroplast, were removed. To further remove possible spurious ASVs [29], we removed any ASV that never reached 0.1% of the reads in any sample and ASVs found in only one sample. With the decontam and phyloseq packages in R v4.3.1 [30–32], we used the prevalence method to identify possible contaminant ASVs based on the four control samples with a threshold of 0.1, removing 49 ASVs and 1.3% of the filtered reads. Analyses excluded control samples and one failed toad sample (Table 1). Unless otherwise stated, analyses were conducted in R on reads rarefied (normalised) to the lowest read depth (14,584 reads per sample).

### Effects of Time and Captivity Status

We first used these data to assess the impacts of captivity status, sampling round, and their interaction on five metrics of bacterial community diversity (Faith’s phylogenetic diversity, richness, Shannon diversity, Bray–Curtis, and Jaccard distance). Alpha diversity metrics were analysed with ANOVAs, with marginal *p*-values calculated in the *car* package [33]. Beta diversity metrics were analysed with permutational multivariate ANOVAs (PERMANOVAs) with adonis2 in the *vegan* package [34], sequentially assessing significance for each predictor. Where relevant, pairwise post-hoc comparisons were conducted with *emmeans* [35]. With these same methods, we replaced sampling round with season (wet or dry) to identify the extent to which temporal patterns are explained by differences in seasonal conditions. For captive toads, we additionally assessed the impact of enclosure on beta diversity metrics with adonis2 with a blocked design, permuting over toad IDs.

### Common and Differentially Abundant Taxa

Alongside detecting statistical differences and similarities among the groups of toads, identifying core (prevalent) taxa and overlapping core communities can further illustrate similarities between wild and captive toads. QIIME2 was used to identify core ASVs in wild and captive toads, defining core as those present in at least 80% of the samples in either group.

Next, we identified specific taxa that differed in relative abundance between wild and captive toads. We used the full, un-rarefied data set to detect differential abundance of bacterial orders (or next lowest identified taxonomic level) between wild and captive toads using ANCOM-BC [36] in QIIME2 with default parameters and false-discovery-rate *p*-value adjustment. Significant bacterial orders are visualised with a heatmap. By default, ANCOM-BC only analyses taxa with at least 10% prevalence. Though ANCOM-BC can only compare two groups, we further used heatmaps to detect temporal groupings of bacterial orders within wild and captive toads (including intake samples), limiting the visualisations to orders present in at least 10% of individuals and representing at least 0.05% of the reads within a captivity group (Supplemental Materials).

### Understanding Drivers of Bacterial Diversity

For captive toads and their intake samples, we assessed stability based on paired Bray–Curtis distances per toad between sampling time points. First, we tested the prediction that communities on individuals were increasingly different from their intake samples over time in captivity (i.e. paired distances between intake and sampling rounds 3, 4, 5, 6) with a linear mixed effects model (LMM; lme4 package; [37]) including toad ID as a random factor. Next, we used a LMM to test the prediction that community stability increased with captivity, such that beta diversity between subsequent sampling rounds decreased with time in captivity (i.e. paired distances of intake and sampling round 3, 3 and 4, 4, and 5, 5 and 6). Conditional *R*^2^ was calculated with the MuMIn package [38]. For qualitative comparison, we also extracted pairwise Bray–Curtis distances within each sampling round for captive toads.

In wild toads, we calculated body condition as mass divided by snout-urostyle length. As ASV richness differed by sampling season (see *Results*), we assessed the correlation of body condition with ASV richness with a linear model including season and the interaction of season and body condition in the model.

To investigate patterns of ASV presence and abundance, which explain community changes over time, we assessed ASV transience (i.e. how consistently present ASVs are in the community) and volatility (i.e. how consistently abundant ASVs are in the community) in captive individuals, as per Ellison et al. [13].

For each ASV per captive toad, including intake samples, we defined transience as the proportion of an individual toad’s samples (proportion of all sampled time points) that an ASV was absent. We then created three groups of transience values based on their average relative abundances in the normalised data set: low, < 0.1%; medium, 0.1–1%; and high abundance, > 1% [13]. Transience was compared between the three abundance groups with a Kruskal–Wallis test and post hoc Dunn’s test (FSA package; [39]).

Volatility was assessed beginning with the unrarefied data set, keeping the top 39 ASVs with total abundance of at least 0.25%, and normalising to the lowest sampling depth of 7,118 reads per sample. For each captive individual, we calculated the mean and standard deviation of the relative abundance (0–1, as 0–100% of the reads) of each ASV. A generalised additive mixed model (GAMM) with the REML method was used to examine the relationship between relative abundance and ASV volatility (standard deviations), with toad ID as a random factor (mgcv package; [40]). Low volatility at low abundances is an expected artifact due to the nature of the index as a standard deviation, and here we focus on patterns of volatility at higher levels of abundance.

## Results

Amplicon sequencing resulted in 6,470,431 bacterial reads in 39,376 ASVs. The final data set prior to normalisation contained 1,606 ASVs in 14,584–66,686 reads per sample. All ASVs were retained in the normalised data set.

### Temporal Patterns of Skin Communities on Wild and Captive Toads

**Fig. 1:**
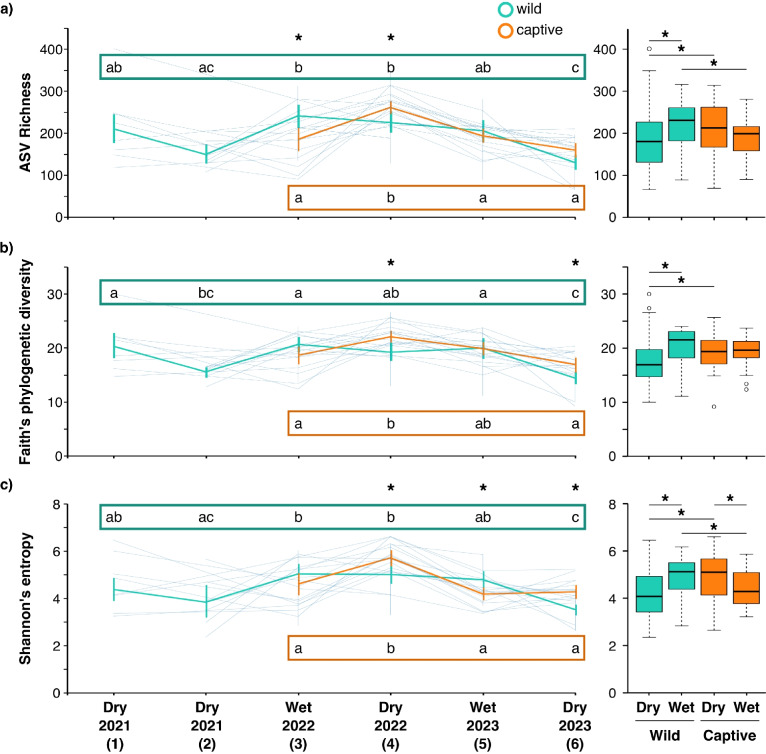
Toad bacterial communities change in alpha diversity over time and based on captivity status. **a**) ASV richness. **b**) Faith’s phylogenetic diversity. **c**) Shannon’s entropy. Left panels depict average values per sampling time point with standard deviations, as well as thinner blue spaghetti plots over time for each individual toad that was tracked throughout its time in captivity. Right panels are boxplots combining multiple years of sampling for wet and dry seasons. Data are colour-coded by captivity status (turquoise = wild, orange = captive). Letters above spaghetti plots (outlined in turquoise) denote significant differences among sampling points within wild toads. Letters below spaghetti plots (outlined in orange) denote significant differences among sampling points within captive toads. Asterisks above spaghetti plots denote time points when wild and captive values differed. Asterisks above boxplots denote significant differences between groups. Spaghetti plots were produced with the q2-longitudinal plug-in in QIIME2 [52]

**Fig. 2:**
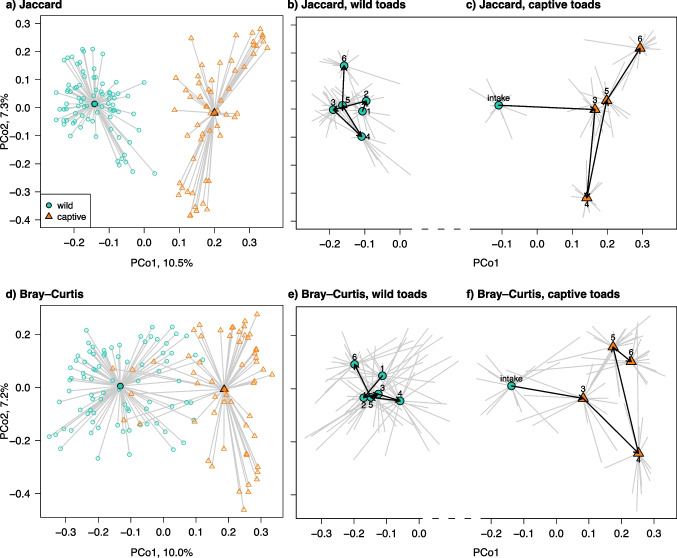
Beta diversity on toad skin differs between captive and wild individuals. (**a**) Jaccard and (**d**) Bray–Curtis centroids and individual points for wild (turquoise circles) and captive (orange triangles) toads. The pattern of change over time differs between wild and captive toads in both Jaccard (**b**, **c**) and Bray–Curtis (**e**, **f**) distances. “Intake” values are values from sampling points 1 & 2 from toads followed throughout their time in captivity. Sampling rounds 1, 2, 4, and 6 were in dry seasons, while 3 and 5 were wet seasons. Arrows follow movement of centroids over the sampling rounds

There was a significant impact of time and its interaction with captivity status in both community structure and composition (Tables 2 and 3; Figs. 1 and 2). Bacteria in wet season samples were more diverse (alpha diversity; Fig. 1) than dry season samples in wild, but not captive, toads. However, the wet season also brought upon lower dispersion (Jaccard distance, F_(1,138)_ = 15.42, *p* = 0.001), with community membership more similar among toads in the wet than dry season. This is especially apparent in visualisations (Fig. 2b, c).

**Table 2:** Impacts of captivity and sampling round on five diversity metrics. Richness, Faith’s phylogenetic diversity, and Shannon’s entropy were analysed with ANOVAs. Jaccard and Bray–Curtis were analysed with adonis2. Bold denotes significance

Diversity metric	Predictor	F	*R*^2^	*P*
ASV richness	Captivity status	F_(1,130)_ = 0.0001		1
	Sampling round	F_(5,130)_ = 14.68		** < 0.0001**
	Captivity * sampling round	F_(3,130)_ = 5.67		**0.001**
Faith’s phylogenetic diversity	Captivity status	F_(1,130)_ = 2.23		0.1
	Sampling round	F_(5,130)_ = 11.87		** < 0.0001**
	Captivity * sampling round	F_(3,130)_ = 4.13		**0.008**
Shannon’s Entropy	Captivity status	F_(1,130)_ = 0.64		0.4
	Sampling round	F_(5,130)_ = 11.97		** < 0.0001**
	Captivity * sampling round	F_(3,130)_ = 6.19		**0.0006**
Jaccard	Captivity status	F_(1,130)_ = 17.39	0.089	**0.001**
	Sampling round	F_(5,130)_ = 6.62	0.169	**0.001**
	Captivity * sampling round	F_(3,130)_ = 5.15	0.079	**0.001**
Bray–Curtis	Captivity status	F_(1,130)_ = 30.93	0.141	**0.001**
	Sampling round	F_(5,130)_ = 7.36	0.168	**0.001**
	Captivity * sampling round	F_(3,130)_ = 7.29	0.100	**0.001**

**Table 3:** Impacts of captivity and season on five diversity metrics. Richness, Faith’s phylogenetic diversity, and Shannon’s entropy were analysed with ANOVAs. Jaccard and Bray–Curtis were analysed with adonis2. Bold denotes significance

Diversity metric	Predictor	F	*R*^2^	*P*
ASV richness	Captivity status	F_(1,136)_ = 0.004		0.9
	Season	F_(1,136)_ = 1.81		0.2
	Captivity * Season	F_(1,136)_ = 8.70		**0.004**
Faith’s phylogenetic diversity	Captivity status	F_(1,136)_ = 0.89		0.3
	Season	F_(1,136)_ = 5.13		**0.03**
	Captivity * Season	F_(1,136)_ = 5.32		**0.02**
Shannon’s Entropy	Captivity status	F_(1,136)_ = 1.63		0.2
	Season	F_(1,136)_ = 0.58		0.4
	Captivity * Season	F_(1,136)_ = 15.56		**0.0001**
Jaccard	Captivity status	F_(1,136)_ = 14.45	0.089	**0.001**
	Season	F_(1,136)_ = 7.52	0.046	**0.001**
	Captivity * Season	F_(1,136)_ = 4.84	0.030	**0.001**
Bray–Curtis	Captivity status	F_(1,136)_ = 24.73	0.141	**0.001**
	Season	F_(1,136)_ = 7.57	0.043	**0.001**
	Captivity * Season	F_(1,136)_ = 7.28	0.041	**0.001**

### Effects of Captivity on Skin Bacteria

Bacterial communities on captive toads did not well represent those on wild toads. At each of the time points when both wild and captive toads were sampled, the communities differed by captivity status in one or more alpha diversity metrics, but there was no regular pattern in direction of change (Fig. 1). Wild and captive toads differed in both beta diversity metrics, accounting for up to 14% of the variation (Tables 2 and 3; Fig. 2). Communities on captive toads also had more similar membership (significantly lower dispersion in Jaccard; F_(1,138)_ = 61.373, *p* = 0.001) than those on wild toads. Captive toads had more similar communities of bacterial orders within sampling rounds than wild toads, evidenced by strong clustering in heatmaps (Fig. S1, S2). Furthermore, changes over time in communities were different between wild and captive toads (Fig. 2), and not all seasonal differences detected in wild toads were found in captive toads (Fig. 1).

Bacterial community composition in captive toads was influenced by captive enclosure, explaining 12–13% of the variation on toads in captivity (Jaccard: F_(2,52)_ = 6.04, *p* = 0.001; Bray–Curtis: F_(2,52)_ = 7.35, *p* = 0.001).

Wild toads had 24 core ASVs, 18 of which were rare with median proportions under 1%, while two were relatively abundant (an uncultured Bacteroidota: *Niabella* and an unknown Actinobacteriota: Micrococcales, with 19% and 21% median proportional abundance, respectively). Toads in captivity had 27 core ASVs, 17 of which were rare. Interestingly, one of the core ASVs with higher relative abundances in wild toads (an uncultured Bacteroidota: *Niabella*) also had a median proportion of 18% in the core community of captive toads. Of the core ASVs, wild and captive toads had 16 overlapping taxa, representing 11.2–92.8% (average 63.4%) of the normalised reads from wild toads and 15.7–88.8% (average 63.1%) of the normalised reads from captive toads.

Sixty-eight bacterial orders differed in abundance between wild and captive toads (Fig. S3), most of which were enriched in captive toads.

**Fig. 3:**
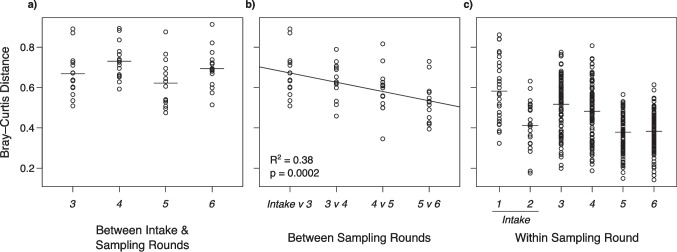
Community stability throughout captivity. (**a**) Within individual toads, Bray–Curtis distances were high between intake and each captive sampling. (**b**) Within individual toads, distance over sampling rounds decreased. (**c**) For comparison, beta diversity between toads, within sampling rounds

Community composition changed after toads were brought into captivity (Fig. 2), with consistently high Bray–Curtis distances between intake samples and the other time points (i.e. no linear pattern, *t* = −0.33, *p* = 0.7; Fig. 3a). Concurrently, bacterial communities became more similar on individual toads over time in captivity (*t* = −4.17, *p* = 0.0002; Fig. 3b).

### Drivers of Diversity

**Fig. 4:**
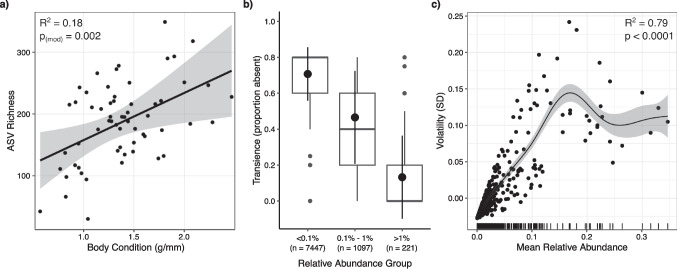
ASV richness, transience, and volatility. (**a**) ASV richness correlates with toad body condition in wild toads. Points are partial residuals, accounting for season. (**b**) ASV transience decreased with increased abundance in captive toads. (**c**) ASV volatility in captive toads fitted with generalised additive mixed model. Points indicate individual partial effects per ASV per toad. ASVs only include the 39 with ≥ 0.25% total relative abundance in the samples. (**a**) and (**c**) include 95% confidence intervals (grey ribbons) and adjusted-*R*^2^ values

In the wild, toads with greater body condition had greater bacterial diversity on their skin (season: *t* = 2.10, *p* = 0.04; body condition: *t* = 2.59, *p* = 0.01; interaction: *t* = −1.77, *p* = 0.08; Fig. 4a).

Low-abundance, rare ASVs had greater transience, flipping in and out of communities more often than higher-abundance ASVs (chi-squared = 1595.1, df = 2, *p* < 0.0001; each pairwise *p* < 0.0001; Fig. 4b). Volatility increased and leveled off with increasing relative abundance, suggesting maintained variability in the more abundant taxa (*F* = 305.5, *p* < 0.0001, adjusted *R*^2^ = 0.793; Fig. 4c).

## Discussion

Seasonal sampling of skin bacteria on wild and captive cane toads revealed patterns of community diversity and stability, providing insights into the extent to which host captivity can reflect community changes in the wild. Results supported our hypotheses that bacterial communities change over time and with seasons, and that toads housed in semi-natural enclosures do not harbour the same communities as wild individuals. Contrary to our second hypothesis, just as skin communities differed between wild and captive toads, so did changes in communities through time. Understanding patterns of host-associated skin communities is imperative to an understanding of the roles that microbes play in host health.

### Microbial Stability

While bacteria on anurans vary throughout ontogeny, skin communities on terrestrial adults reach a relatively steady state [3, 10]. Regardless, this study and others observed temporal changes impacted by seasons and weather [3, 4, 11]. Across 2 years of sampling, we found differing diversity within a season among years, particularly among the dry seasons. Just as Brazil’s tree frogs (Hylidae and Phyllomedusidae) have greater skin bacterial richness at wetter sites [41], and drought can lead to dysbiosis on Brazil’s pumpkin toadlets (*Brachycephalus rotenbergae*) [17], wet season brought increased alpha diversity and more similar communities among toads (beta diversity). Perhaps this reflects greater diversity in environmental bacterial reservoirs during the wet season or wet season conditions enabling more taxa to survive on toad hosts. Importantly, sampling round explained more variation than season, because communities did not return to a “dry” or “wet” season composition but rather continuously changed through time. The association between body condition and diversity on wild toads supports others’ findings [18] that intrinsic host factors modulate bacterial colonisation and success.

Broader changes within bacterial communities over time can be attributed to shifts in individual taxa. In captive toads, bacteria with greater relative abundance had greater retention in communities across sampling rounds (in agreement with [13]). On the other hand, less abundant taxa were more transient. The leveling-off of volatility for abundant taxa suggests a threshold for stability in abundance. When Ellison et al. [13] found low volatility in high-abundant taxa on *R. sierrae*, they had re-sampled individuals in the active season in one or two years. Repeat samples over shorter time periods may have led to their detection of greater microbial stability (i.e. lower volatility) than would be present in sampling spread out over seasons and years.

Cane toads experience a variety of ecosystems in their native and invaded ranges, from wet and wet–dry tropics to drier seasonal subtropics [42, 43]. Cane toads’ reduced susceptibility to chytridiomycosis (particularly in adults, [44, 45]) and non-threatened status may result in less attention among microbiome studies (but see [6, 12, 46]). However, their wide distribution makes cane toads an interesting model to investigate bacterial variation, such as the roles of intrinsic and ecological factors on community assemblage and composition. Furthermore, examining the toad’s invasion into diverse regions over the last 100 years from a microbiological prospective provides an interesting direction for understanding ecological requirements and flexibility in invasion biology.

### Captivity Representing Wild Toads

Despite captive toads being held in semi-natural conditions, exposed to the same natural climatic variation as the wild toads, their skin communities and patterns over time differed from those of wild toads. Free-ranging toads would have experienced more variable environmental conditions, including different soil types and water sources. A lack of diverse bacterial reservoirs likely led to the increased stability found on captive toads over time. While stability may be expected for abundant taxa, maintenance of rare, under-represented taxa relies on reservoirs and complex environments [14, 15, 47]. Captive toads in this experiment often harboured similarly rich communities as those on wild toads, but membership (Jaccard) diverged based on captivity status, beginning with a large shift in membership after intake. Though the overlapping core taxa between captive and wild toads made up a high proportion of reads in the samples, even the prevalent bacteria did not comprise similar communities, in structure and composition (Fig. S4), between wild and captive toads.

In captivity, toads have limited ecological interactions, limited in exposure to diverse habitats and bacterial reservoirs, but also protected from parasitic interactions and potentially limited in breadth of food options, which may directly or indirectly impact their skin microbiomes [14, 18, 47, 48]. Besides containing the toads to a small area, captive conditions in this study also included artificial watering from above, in contrast with natural freshwater available to the wild population, which could especially impact bacteria on captive toads in the dry season. An important limitation to this study was the uneven sampling design, comparing resampled captive individuals with unique wild individuals. Interestingly, we found greater shifts in community membership in captive than wild toads over time, despite reduced intra-individual variation in composition (Bray–Curtis) with time. Determining whether high intra-individual variation is natural in this system rather than a result of captivity is an important step in understanding this species’ ecology. Cane toads in this region move a considerable distance each day [49], making them difficult to re-sample in the wild [19]. However, with the aid of radio-tracking and incorporation of habitat data, further work could reveal the impacts of diverse habitats and whether time and distance traversed through variable vegetation types affect symbiotic bacterial diversity on toads across their widespread distribution [49, 50].

### Ecological Context

One challenge to understanding skin communities is separating host-specific effects from environmental factors. Concepts such as core and keystone taxa attempt to explain which microbes are important to hosts, but their functional significance largely remains unresolved. An understanding of keystone microbes is emerging in the soil, plant, and human literature [51], but is slow to gain traction in studies of wildlife hosts. Though putative keystone taxa can be identified computationally, finding empirical evidence of their impact is more complex, and this step is outside the scope of many wildlife studies. An exception is probiotic research to combat amphibian chytridiomycosis [9].

Our study provides insight into the relative role of the environment in the maintenance of toad skin bacterial communities. For example, wild and captive toads harboured a handful of common core taxa comprising a large proportion of the community on most individuals (> 63% relative abundance). Toads entering captivity largely retained these taxa over the course of years, concurrent with wild toads also possessing those taxa in high abundance, suggesting intrinsic drivers for the retention of core bacteria. Possibly, some or all of these core microbes are ubiquitous in the environment and repeatedly recolonise toad skin (as detected for some core taxa on amphibians; [47]). However, a working hypothesis could be that most persistent core microbes are favoured by intrinsic characteristics of the toad skin (i.e. selected for by the host; [47]).

Some studies have evaluated overlapping core communities on host populations across a large landscape to identify the relative importance of intrinsic versus environmental drivers of bacterial assembly on amphibians. The absence of overlapping core taxa on invasive American bullfrogs (*Lithobates catesbeianus*) on three continents suggests a strong impact of local environment on those communities [16] and may be explained by functional replacements (i.e. functional core). In contrast, cane toads from across Australia had overlapping core taxa representing a relatively large proportion of their communities [12], notwithstanding the possibility that there has also been functional replacement of some microbes across this landscape. Unsurprisingly, the existence of core taxa is dependent on the system and may provide insight into the manner of interaction between a host and its skin community.

Furthermore, an unknown portion of the skin microbiome is simply an artifact of the animal’s recent exposure to microbes in the environment. Given that 13% of variation in captive toads was determined by their enclosure, despite the enclosures being side-by-side, suggests that a sizable portion of community variation is due to random factors allowing drift despite similarities in diet, weather, etc. Indeed, one study found up to 40% of bacteria on red-backed salamanders (*Plethodon cinereus*) were driven by neutral (random) processes [47]. Additionally, studies like ours that focus on trends in bacterial diversity rarely include data on bacterial biomass or total abundance, which may not align with diversity metrics such as richness. Time points with high richness may even have reduced biomass, with greater resource availability allowing entry to more transient taxa. Invasion of pathogens into the system can also exploit and exhaust resources, outcompeting resident bacteria and consequently reducing richness and biomass of the resident taxa.

To uncover the roles of host-promoted microbes that meet the ecological and physiological requirements of hosts, we need a combination of approaches to distinguish transient microbes from those with functional interactions with the host and discern patterns in microbial abundance through time. Shotgun metagenomics could be used to detect functional replacement in natural systems, with challenge experiments revealing functional importance of microbes on animal hosts. Detailed understanding of skin chemistry (secretions) and its impact on microbes would clarify mechanisms of those selective processes. Pairing quantitative PCR with culturing would help describe stability of microbial abundance. A full understanding of both the composition and roles of skin microbiomes will require experimental manipulations and examination of organisms in natural systems. The transcontinental distribution of cane toads could be exploited to provide important insight into host–microbiome interactions.

## Supplementary Information

Below is the link to the electronic supplementary material.

Supplementary file1 (PDF 3086 KB)

## Data Availability

Amplicon sequence data are available on NCBI’s Sequence Read Archive (BioProject accession: PRJNA1199167).

## References

[1] Rosshart SP, Vassallo BG, Angeletti D et al (2017) Wild mouse gut microbiota promotes host fitness and improves disease resistance. Cell 171:1015–1028. 10.1016/j.cell.2017.09.016 29056339 10.1016/j.cell.2017.09.016 PMC6887100

[2] Harris-Tryon TA, Grice EA (2022) Microbiota and maintenance of skin barrier function. Science 376:940–945. 10.1126/science.abo0693 35617415 10.1126/science.abo0693

[3] Longo AV, Savage AE, Hewson I, Zamudio KR (2015) Seasonal and ontogenetic variation of skin microbial communities and relationships to natural disease dynamics in declining amphibians. R Soc Open Sci 2:140377. 10.1098/rsos.140377 26587253 10.1098/rsos.140377 PMC4632566

[4] Douglas AJ, Hug LA, Katzenback BA (2021) Composition of the North American wood frog (*Rana**sylvatica*) bacterial skin microbiome and seasonal variation in community structure. Microb Ecol 81:78–92. 10.1007/s00248-020-01550-5 32613267 10.1007/s00248-020-01550-5

[5] Jones KR, Walke JB, Becker MH et al (2021) Time in the laboratory, but not exposure to a chytrid fungus, results in rapid change in spring peeper (*Pseudacris**crucifer*) skin bacterial communities. Ichthyol Herpetol 109:75–83. 10.1643/h2020077

[6] Weitzman CL, Brown GP, Gibb K, Christian K (2024) Cutaneous shedding in amphibians causes shifts in bacterial microbiomes. Integr Zool In Press. 10.1111/1749-4877.12858 10.1111/1749-4877.12858 PMC12235345 38897983

[7] Moeller AH, Ivey K, Cornwall MB et al (2020) The lizard gut microbiome changes with temperature and is associated with heat tolerance. Appl Environ Microbiol 86:e01181-e1220. 10.1128/AEM.01181-20 32591376 10.1128/AEM.01181-20 PMC7440792

[8] Meyer EA, Cramp RL, Bernal MH, Franklin CE (2012) Changes in cutaneous microbial abundance with sloughing: possible implications for infection and disease in amphibians. Dis Aquat Org 101:235–242. 10.3354/dao02523 10.3354/dao02523 23324420

[9] Rebollar EA, Martínez-Ugalde E, Orta AH (2020) The amphibian skin microbiome and its protective role against chytridiomycosis. Herpetologica 76:167–177. 10.1655/0018-0831-76.2.167

[10] Prest TL, Kimball AK, Kueneman JG, McKenzie VJ (2018) Host-associated bacterial community succession during amphibian development. Mol Ecol 27:1992–2006. 10.1111/mec.14507 29411448 10.1111/mec.14507

[11] Varela BJ, Lesbarrères D, Ibáñez R, Green DM (2018) Environmental and host effects on skin bacterial community composition in Panamanian frogs. Front Microbiol 9:298. 10.3389/fmicb.2018.00298 29520260 10.3389/fmicb.2018.00298 PMC5826957

[12] Weitzman CL, Kaestli M, Rose A et al (2023) Geographic variation in bacterial assemblages on cane toad skin is influenced more by local environments than by evolved changes in host traits. Biol Open 12:bio059641. 10.1242/bio.059641 36745034 10.1242/bio.059641 PMC9932784

[13] Ellison S, Knapp R, Vredenburg V (2021) Longitudinal patterns in the skin microbiome of wild, individually marked frogs from the Sierra Nevada. California ISME Commun 1:45. 10.1038/s43705-021-00047-7 37938625 10.1038/s43705-021-00047-7 PMC9723788

[14] Harrison XA, Price SJ, Hopkins K et al (2019) Diversity-stability dynamics of the amphibian skin microbiome and susceptibility to a lethal viral pathogen. Front Microbiol 10:2883. 10.3389/fmicb.2019.02883 31956320 10.3389/fmicb.2019.02883 PMC6951417

[15] Loudon AH, Woodhams DC, Parfrey LW et al (2014) Microbial community dynamics and effect of environmental microbial reservoirs on red-backed salamanders (*Plethodon**cinereus*). ISME J 8:830–840. 10.1038/ismej.2013.200 24335825 10.1038/ismej.2013.200 PMC3960541

[16] Kueneman JG, Bletz MC, McKenzie VJ et al (2019) Community richness of amphibian skin bacteria correlates with bioclimate at the global scale. Nat Ecol Evol 3:381–389. 10.1038/s41559-019-0798-1 30778181 10.1038/s41559-019-0798-1

[17] Buttimer S, Moura-Campos D, Greenspan SE et al (2024) Skin microbiome disturbance linked to drought-associated amphibian disease. Ecol Lett 27:e14372. 10.1111/ele.14372 38288868 10.1111/ele.14372

[18] Longo AV, Zamudio KR (2017) Temperature variation, bacterial diversity and fungal infection dynamics in the amphibian skin. Mol Ecol 26:4787–4797. 10.1111/mec.14220 28664981 10.1111/mec.14220

[19] Hudson CM, Brown GP, Shine R (2017) Effects of toe-clipping on growth, body condition, and locomotion of cane toads (*Rhinella**marina*). Copeia 105:257–260. 10.1643/CE-16-564

[20] Christian K, Weitzman C, Rose A et al (2018) Ecological patterns in the skin microbiota of frogs from tropical Australia. Ecol Evol 8:10510–10519. 10.1002/ece3.4518 30464823 10.1002/ece3.4518 PMC6238143

[21] Caporaso JG, Lauber CL, Walters WA et al (2011) Global patterns of 16S rRNA diversity at a depth of millions of sequences per sample. Proc Natl Acad Sci USA 108:4516–4522. 10.1073/pnas.1000080107 20534432 10.1073/pnas.1000080107 PMC3063599

[22] Apprill A, McNally S, Parsons R, Weber L (2015) Minor revision to V4 region SSU rRNA 806R gene primer greatly increases detection of SAR11 bacterioplankton. Aquat Microb Ecol 75:129–137. 10.3354/ame01753

[23] Parada AE, Needham DM, Fuhrman JA (2016) Every base matters: assessing small subunit rRNA primers for marine microbiomes with mock communities, time series and global field samples. Environ Microbiol 18:1403–1414. 10.1111/1462-2920.13023 26271760 10.1111/1462-2920.13023

[24] Bolyen E, Rideout JR, Dillon MR et al (2019) Reproducible, interactive, scalable and extensible microbiome data science using QIIME 2. Nat Biotechnol 37:852–857. 10.1038/s41587-019-0209-9 31341288 10.1038/s41587-019-0209-9 PMC7015180

[25] Callahan BJ, McMurdie PJ, Rosen MJ et al (2016) DADA2: High-resolution sample inference from Illumina amplicon data. Nat Methods 13:581–583. 10.1038/nmeth.3869 27214047 10.1038/nmeth.3869 PMC4927377

[26] Pedregosa F, Varoquaux G, Gramfort A et al (2011) Scikit-learn: Machine learning in Python. J Mach Learn Res 12:2825–2830

[27] Quast C, Pruesse E, Yilmaz P et al (2012) The SILVA ribosomal RNA gene database project: improved data processing and web-based tools. Nucleic Acids Res 41:D590–D596. 10.1093/nar/gks1219 23193283 10.1093/nar/gks1219 PMC3531112

[28] Yilmaz P, Parfrey LW, Yarza P et al (2014) The SILVA and “all-species living tree project (LTP)” taxonomic frameworks. Nucleic Acids Res 42:D643–D648. 10.1093/nar/gkt1209 24293649 10.1093/nar/gkt1209 PMC3965112

[29] Reitmeier S, Hitch TCA, Treichel N et al (2021) Handling of spurious sequences affects the outcome of high-throughput 16S rRNA gene amplicon profiling. ISME Commun 1:31. 10.1038/s43705-021-00033-z 37938227 10.1038/s43705-021-00033-z PMC9723555

[30] McMurdie PJ, Holmes S (2013) phyloseq: an R package for reproducible interactive analysis and graphics of microbiome census data. PLoS One 8:e61217. 10.1371/journal.pone.0061217 23630581 10.1371/journal.pone.0061217 PMC3632530

[31] Davis NM, Proctor DM, Holmes SP et al (2018) Simple statistical identification and removal of contaminant sequences in marker-gene and metagenomics data. Microbiome 6:226. 10.1186/s40168-018-0605-2 30558668 10.1186/s40168-018-0605-2 PMC6298009

[32] R Core Team (2023) R: A Language and Environment for Statistical Computing

[33] Fox J, Weisberg S (2019) An R Companion to Applied Regression, 3rd edn. Sage, Thousand Oaks CA

[34] Oksanen J, Simpson GL, Blanchet FG et al (2024) vegan: Community Ecology Package

[35] Lenth R (2024) emmeans: Estimated marginal means, aka least-squares means. R Package Vers 1(10):2

[36] Lin H, Peddada SD (2020) Analysis of compositions of microbiomes with bias correction. Nat Commun 11:3514. 10.1038/s41467-020-17041-7 32665548 10.1038/s41467-020-17041-7 PMC7360769

[37] Bates D, Mächler M, Bolker B, Walker S (2015) Fitting linear mixed-effects models using lme4. J Stat Softw 67:1–48. 10.18637/jss.v067.i01

[38] Bartoń K (2024) MuMIn: Multi-Model Inference. R Package Vers 1(48):4

[39] Ogle DH, Doll JC, Wheeler P, Dinno A (2023) FSA: Simple Fisheries Stock Assessment Methods. R Package Version 095 https://CRANR-project.org/package=FSA

[40] Wood SN (2011) Fast stable restricted maximum likelihood and marginal likelihood estimation of semiparametric generalized linear models. J R Stat Soc B 73:3–36. 10.1111/j.1467-9868.2010.00749.x

[41] Ruthsatz K, Lyra ML, Lambertini C et al (2020) Skin microbiome correlates with bioclimate and Batrachochytrium dendrobatidis infection intensity in Brazil’s Atlantic Forest treefrogs. Sci Rep 10:22311. 10.1038/s41598-020-79130-3 33339839 10.1038/s41598-020-79130-3 PMC7749163

[42] Zug GR, Zug PB (1979) The marine toad, *Bufo marinus*: a natural history resume of native populations. Smithsonian Contributions to Zoology

[43] IUCN (2020) The IUCN Red List of Threatened Species. Version 2020–1.

[44] Poorten TJ, Rosenblum EB (2016) Comparative study of host response to chytridiomycosis in a susceptible and a resistant toad species. Mol Ecol 25:5663–5679. 10.1111/mec.13871 27696594 10.1111/mec.13871

[45] Brannelly LA, Martin G, Llewelyn J et al (2018) Age-and size-dependent resistance to chytridiomycosis in the invasive cane toad *Rhinella**marina*. Dis Aquat Org 131:107–120. 10.3354/dao03278 10.3354/dao03278 30460917

[46] Abarca JG, Zuniga I, Ortiz-Morales G et al (2018) Characterization of the skin microbiota of the cane toad *Rhinella**cf*. *marina* in Puerto Rico and Costa Rica. Front Microbiol 8:2624. 10.3389/fmicb.2017.02624 29354109 10.3389/fmicb.2017.02624 PMC5760547

[47] Loudon AH, Venkataraman A, Van Treuren W et al (2016) Vertebrate hosts as islands: Dynamics of selection, immigration, loss, persistence, and potential function of bacteria on salamander skin. Front Microbiol 7:333. 10.3389/fmicb.2016.00333 27014249 10.3389/fmicb.2016.00333 PMC4793798

[48] Risely A, Byrne PG, Hoye BJ, Silla AJ (2024) Dietary carotenoid supplementation has long-term and community-wide effects on the amphibian skin microbiome. Mol Ecol 33:e17203. 10.1111/mec.17203 37962103 10.1111/mec.17203

[49] Shine R, Alford RA, Blennerhasset R et al (2021) Increased rates of dispersal of free-ranging cane toads (*Rhinella**marina*) during their global invasion. Sci Rep 11:23574. 10.1038/s41598-021-02828-5 34876612 10.1038/s41598-021-02828-5 PMC8651681

[50] Becker CG, Longo AV, Haddad CFB, Zamudio KR (2017) Land cover and forest connectivity alter the interactions among host, pathogen and skin microbiome. Proc R Soc B 284:20170582. 10.1098/rspb.2017.0582 28835551 10.1098/rspb.2017.0582 PMC5577473

[51] Banerjee S, Schlaeppi K, van der Heijden MG (2018) Keystone taxa as drivers of microbiome structure and functioning. Nat Rev Microbiol 16:567–576. 10.1038/s41579-018-0024-1 29789680 10.1038/s41579-018-0024-1

[52] Bokulich NA, Dillon MR, Zhang Y et al (2018) q2-longitudinal: Longitudinal and paired-sample analyses of microbiome data. Systems 3:10.1128/msystems.00219-18. 10.1128/mSystems.00219-18 PMC6247016 30505944

