# Supplementary material for: Differential Temporal Shifts in Skin Bacteria on Wild and Captive Toads
Source: Microb Ecol. 2025 Apr 29;88(1):35. doi: 10.1007/s00248-025-02537-w (PMC12040999; doi:10.1007/s00248-025-02537-w)
Supplement: Supplementary file 1 — Supplementary file1 (PDF 3086 KB) [file 248_2025_2537_MOESM1_ESM.pdf]

Supplemental Materials

Differential Temporal Shifts in Skin Bacteria on Wild and Captive Toads

Chava L. Weitzman[1], Kimberley Day[1], Gregory P. Brown[2], Karen Gibb[1], Keith Christian[1]

[1] Research Institute for the Environment and Livelihoods, CDU, Casuarina, NT, Australia

[2] School of Natural Sciences, Macquarie University, Sydney, NSW, Australia

Corresponding author: Chava Weitzman, weitzman.chava@gmail.com

Microbial Ecology

Wild Toads

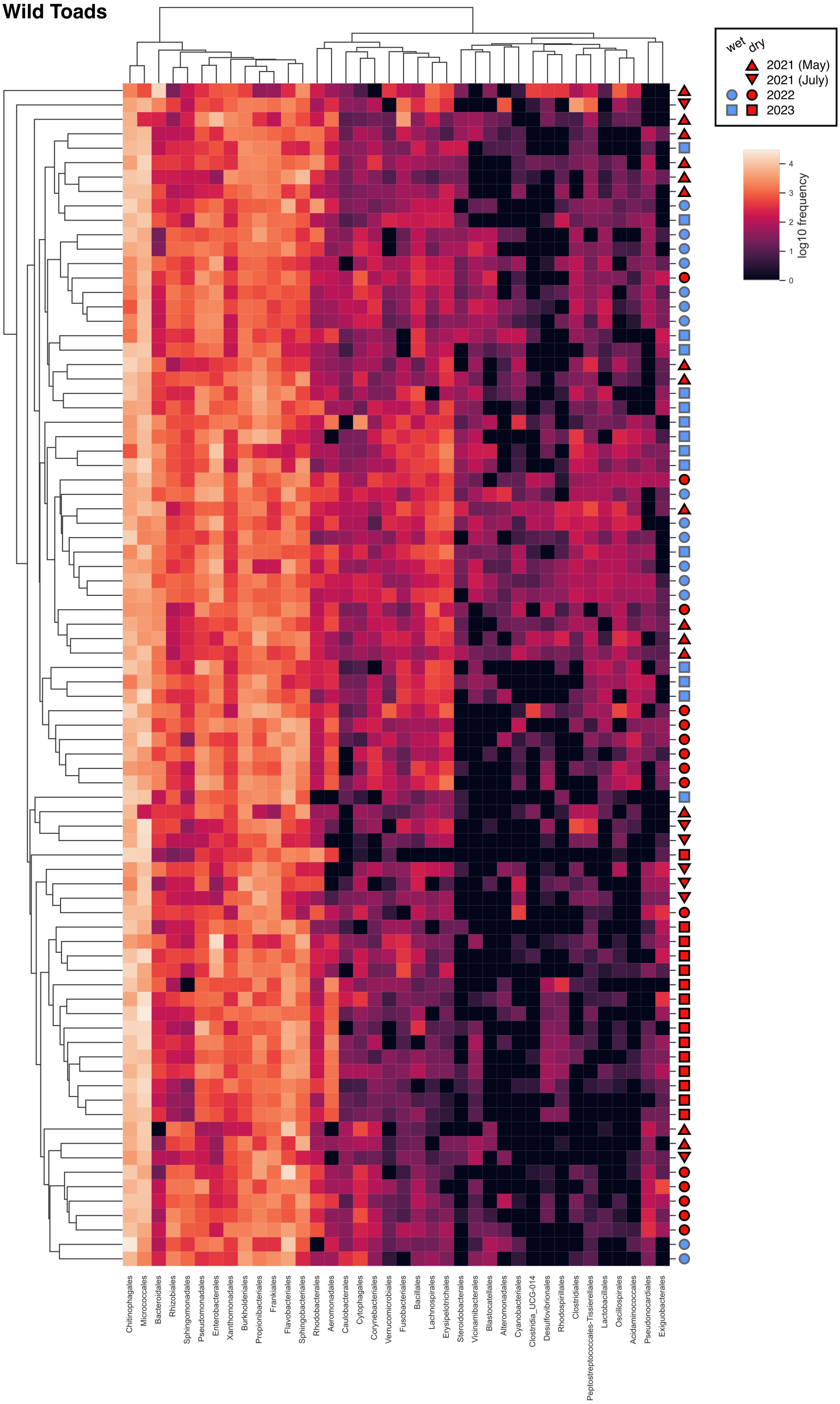

Fig. S1 Heatmap of relatively abundant bacterial orders in wild-caught toads. Symbol next to each row identifies sampling year (shape) and season (colour). Heatmap was made in QIIME2. Dendrograms are based on Euclidean distances

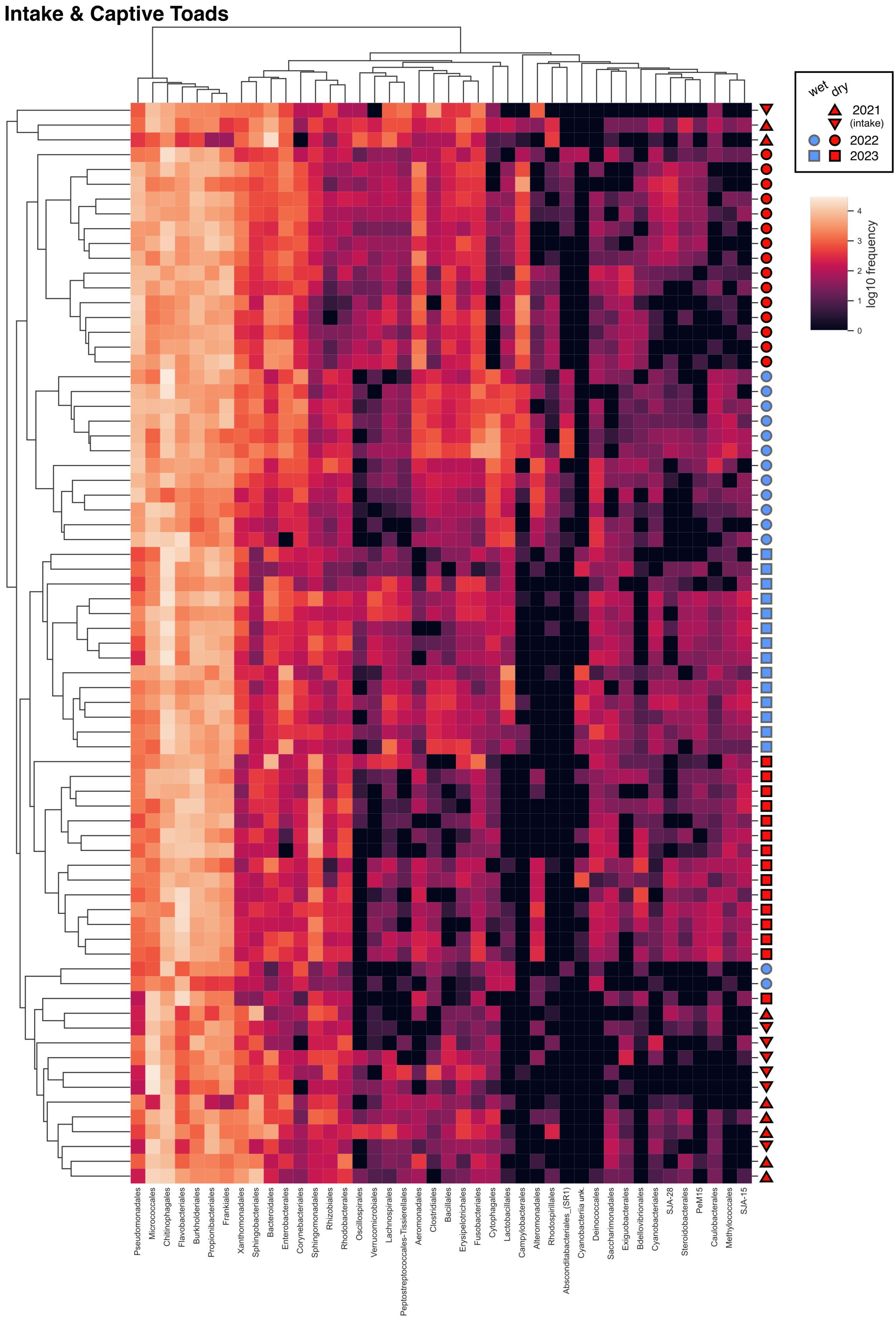

Fig. S2 Heatmap of relatively abundant bacterial orders in captive toads, including intake samples. Symbol next to each row identifies sampling year (shape) and season (colour). Heatmap was made in QIIME2. Dendrograms are based on Euclidean distances

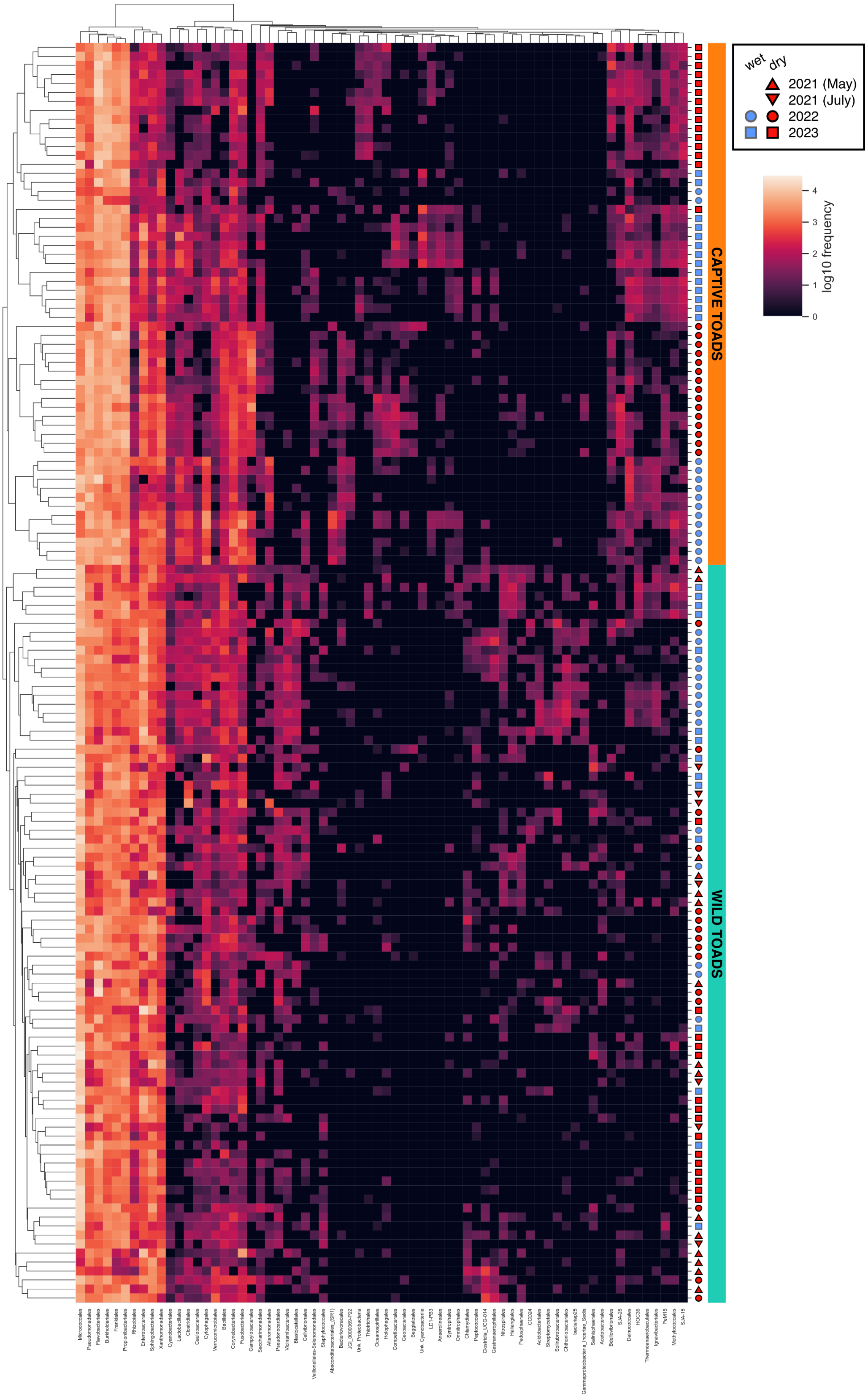

Fig. S3 Heatmap of differentially abundant bacterial orders between wild and captive toads. Symbol next to each row identifies sampling year (shape) and season (colour). Heatmap was made in QIIME2. Dendrograms are based on Euclidean distances

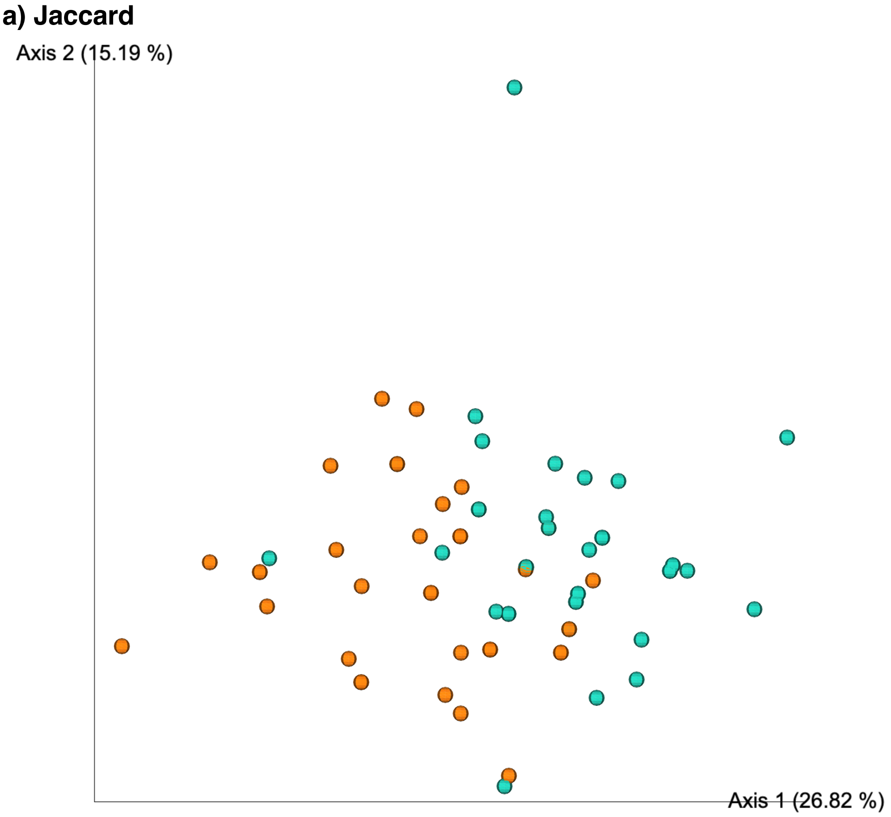

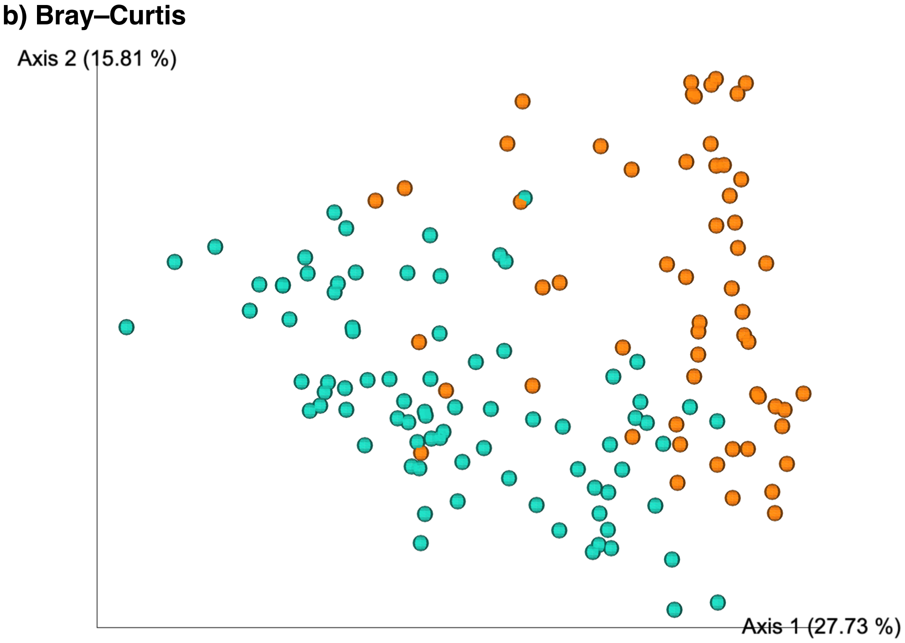

Fig. S4 Principal coordinates analysis including only prevalent ASVs (20 ASVs present in at least 80% of all samples), rarefied to 5,357 reads per sample. Points colour-coded by captivity status (turquoise = wild; orange = captive)
